# Rapid Chemical Profiling of *Filipendula ulmaria* Using CPC Fractionation, 2-D Mapping of ^13^C NMR Data, and High-Resolution LC–MS

**DOI:** 10.3390/molecules28176349

**Published:** 2023-08-30

**Authors:** Steve Thomas Pannakal, Joan Eilstein, Jane Hubert, Alexis Kotland, Arpita Prasad, Amelie Gueguiniat-Prevot, Franck Juchaux, Floriane Beaumard, Ganapaty Seru, Sherluck John, Dhimoy Roy

**Affiliations:** 1 Advanced Research, L’Oréal Research and Innovation India, Bearys Global Research Triangle, Whitefield Ashram Road, Bangalore 560067, India; 2 Advanced Research, L’Oréal Research and Innovation, 1 Avenue Eugène Schueller, 93600 Aulnay-Sous-Bois, France; 3 NatExplore SAS, 25 La Chute des Eaux, 51140 Prouilly, France; 4 Pharmacognosy and Phytochemistry Division, Gitam Institute of Pharmacy, Gitam University, Visakhapatnam 530045, India; 5 L’Oréal India Pvt Ltd., Research & Innovation, 7th Floor, Universal Majestic, Ghatkopar—Mankhurd Link Road, Chembur, Mumbai 400071, India

**Keywords:** *Filipendula ulmaria*, centrifugal partition chromatography, NMR-based dereplication, liquid chromatography mass spectrometry, epidermal barrier renewal

## Abstract

*Filipendula ulmaria*, commonly known as meadowsweet, is a wild herbaceous flowering plant that is widely distributed in Europe. A range of salicylic acid derivatives and flavonol glycosides have been previously associated with the antirheumatic and diuretic properties of *F. ulmaria*. In the present work, a hydroalcoholic extract from *F. ulmaria* aerial parts was extensively profiled using an efficient NMR-based dereplication strategy. The approach involves the fractionation of the crude extract by centrifugal partition chromatography (CPC), ^13^C NMR analysis of the fractions, 2D-cluster mapping of the entire NMR dataset, and, finally, structure elucidation using a natural metabolite database, validated by 2D NMR data interpretation and liquid chromatography coupled with mass spectrometry. The chemodiversity of the aerial parts was extensive, with 28 compounds unambiguously identified, spanning various biosynthetic classes. The *F. ulmaria* extract and CPC fractions were screened for their potential to enhance skin epidermal barrier function and skin renewal properties using in vitro assays performed on Normal Human Epidermal Keratinocytes. Fractions containing quercetin, kaempferol glycosides, ursolic acid, pomolic acid, naringenin, *β*-sitosterol, and Tellimagrandins I and II were found to upregulate genes related to skin barrier function, epidermal renewal, and stress responses. This research is significant as it could provide a natural solution for improving hydration and skin renewal properties.

## 1. Introduction

*Filipendula ulmaria* (L.) Maxim., syn. *Spiraea ulmaria* L. (Meadowsweet) is a wild herbaceous flower belonging to the Rosaceae family and is largely distributed on wet European roads [1,2,3]. The medicinal use of *F. ulmaria* dates back to the late 16th and 17th centuries [4] and has been well-documented in the British Herbal Pharmacopoeia [5,6] as a stomachic, mild urinary antiseptic, antirheumatic, and antacid. In addition, the British Herbal Compendium [7] describes the action of the *Filipendula* herb as anti-inflammatory. The herb and its flowers have been traditionally used as a diuretic and antirheumatic in the treatment of inflammatory diseases. A number of secondary metabolites belonging to phenolic and flavonol glycosides, namely spiraeoside, hyperoside, rutoside, xyloglycoside of methyl salicylate, and the rare salicylic aldehyde, have been previously reported in *Filipendula* species [8,9,10]. These secondary metabolites and related phytochemical structures could be responsible for the biological properties described above. Interestingly, recent studies have reported that α-hydroxy acids, salicylic acid, and its derivatives are responsible for improving skin hydration by gently peeling the corneocytes of the upper epidermis and softening the skin while displaying a significant decrease in stratum corneum cohesion as well as minimally disrupting the skin barrier to water diffusion [11,12,13]. The aim of the present study is to delve deeper into the chemical profile of *F. ulmaria* with a particular focus on its ability to improve skin barrier functions. The development of modern analytical platforms based on nuclear magnetic resonance (NMR) or high-resolution liquid mass spectrometry (LC–MS) have enabled faster detection, identification, and quantification of chemically diverse natural products [14,15,16]. To support the chemical profiling of complex extracts, many dereplication strategies involving computer tools and metabolite databases have also been deployed [17,18], mainly to overcome the repetitive isolation of known compounds and accelerate the identification process [19,20,21,22]. Here, a unique dereplication workflow was used to chemically profile *F. ulmaria* aerial parts, combining centrifugal partition chromatography (CPC), NMR, hierarchical clustering analysis (HCA), and a natural metabolite database [23]. CPC is a rapid liquid–liquid separation technique that enables the fractionation of complex metabolite mixtures across a large polarity range without any loss of biomass and with a high injection capacity at the multi-gram scale. The fraction series produced by CPC is directly analyzed by ^13^C NMR. Subsequently, the complete NMR dataset is aligned in a unified table and submitted to HCA to highlight statistical correlations between groups of ^13^C peaks detected in successive fractions, corresponding to the carbon skeletons of *F. ulmaria* metabolites. Metabolite identification is performed with the help of an NMR database dedicated to small natural molecules, proposing chemical structures that potentially match with the NMR data clusters observed on the HCA heatmap. Database proposals are rigorously validated or reoriented towards the correct solution by the manual interpretation of 2D NMR spectra. This approach provides a detailed chemical profile without discrimination between chemical classes and without biomass loss, which means that 100% of the starting extract mass can be recovered at the end of the identification process under the form of a well-characterized fraction series. One can also note that the mass quantities obtained by CPC for each fraction are sufficiently high to perform biological tests in parallel. Over the last years, this procedure has demonstrated its robustness through applications on natural extracts of various origins including terrestrial plants, microalgae and macroalgae, or cultured cell extracts of a plant or microbial species [24,25,26,27,28,29,30]. This identification process was completed by high-resolution LC–MS analysis of the crude extract. The fractions were then screened for their potential to improve skin epidermal barrier function and skin-renewal properties using appropriate in vitro assays.

## 2. Results and Discussion

### 2.1. Chemical Profiling of the Filipendula ulmaria Extract

The chemical profile of the hydroalcoholic extract of *F. ulmaria* aerial parts was deciphered using a dereplication strategy named “CARAMEL” (CARActérisation des MELanges in French, for mixture characterization), which is based on a metabolomic workflow combining centrifugal partition chromatography, nuclear magnetic resonance, and computational treatments [23]. This strategy enables the direct identification of simplified mixtures of secondary metabolites (CPC fractions) without the need to purify individual constituents.

**Figure 1:**
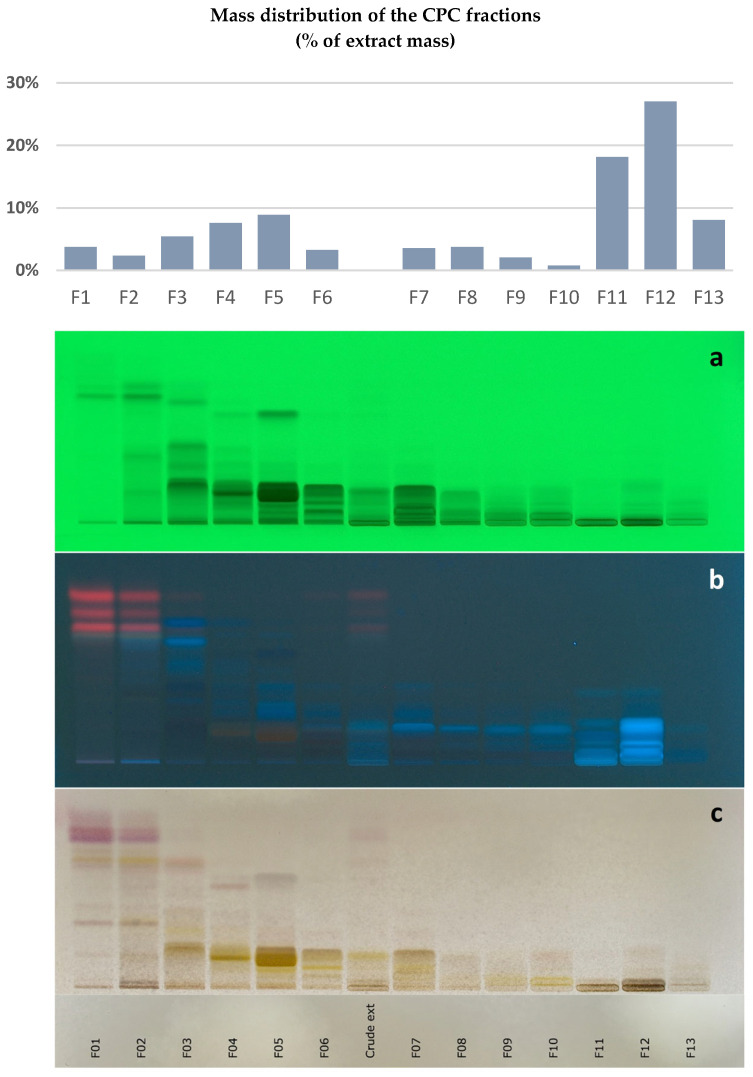
Mass distribution and HPTLC profile of the 13 CPC fractions (F1–F13 and crude extract—(**a**) 254 nm; (**b**) 366 nm; (**c**) Visible after vanillin/H_2_SO_4_ reagent spraying).

As a first step, the *F. ulmaria* extract was fractionated by CPC to produce a series of fractions without a loss of biomass during separation. A biphasic solvent system of medium polarity composed of methyl *ter*-butyl ether, acetonitrile, and water (4/1/5, *v*/*v*) was selected. This system was employed in ascending mode to tentatively separate *F. ulmaria*-specialized metabolites during a single elution step while retaining the most hydrophilic compounds (mainly simple sugars) in the CPC column. A total of 13 final fractions were obtained. Their high-performance thin-layer chromatography (HPTLC) profile is demonstrated in Figure 1.

**Figure 2:**
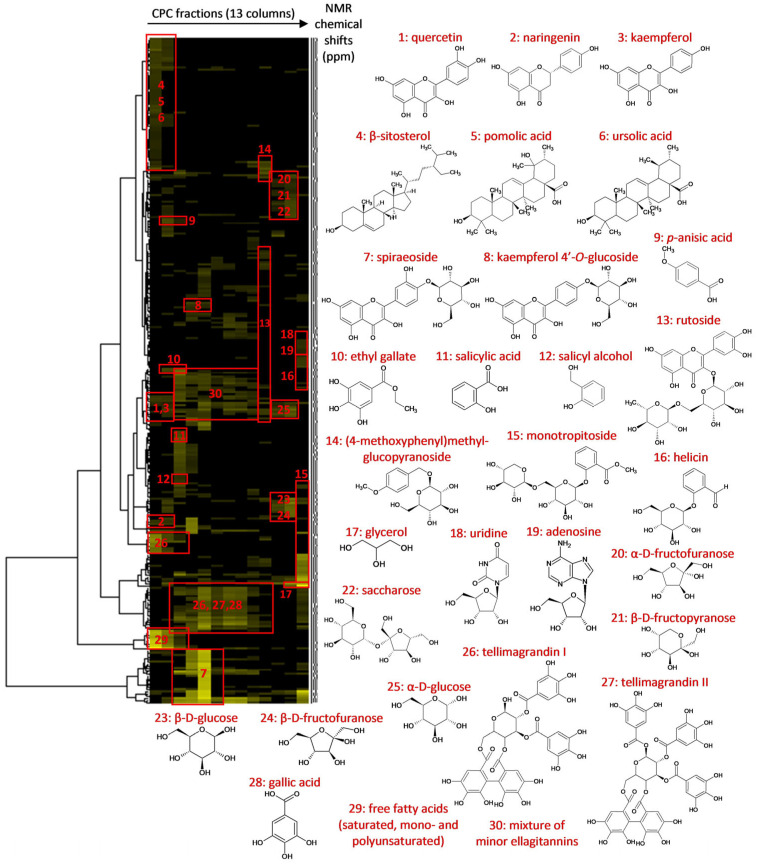
Hierarchical clustering analysis of ^13^C NMR signals detected in the CPC fractions of the *Filipendula ulmaria* extract and identification of 28 secondary metabolites.

Total recovery of the *F. ulmaria* extract mass over fractionation was ~95%. Fractions F01 to F10, which were recovered during the elution step, represented 41.4% of the extract mass and exhibited a very high chemical diversity, as revealed by HPTLC. The most polar fractions, F11–F13, obtained at the end of the fractionation process by the extrusion of the CPC column, represented 53.2% of the extract mass (*w*/*w*). In the second step, all CPC fractions were directly analyzed by 1D and 2D NMR. The mass of each fraction was largely sufficient to achieve NMR analyses (with 15 mg each, except 8 mg for fraction F10) while keeping aside fractions for biological evaluation. Automatic peak picking was performed on ^13^C NMR spectra, and the collected peaks were aligned across the fraction series using a bucketing script. The resulting table was made of 13 columns corresponding to the CPC fractions and 307 rows corresponding to the chemical shift buckets (Δ 0.3 ppm), for which a ^13^C peak was detected in at least one fraction. This table was subjected to HCA for the recognition of similarities between groups of ^13^C NMR peaks detected in adjacent CPC fractions. In this way, ^13^C NMR peaks belonging to the same compounds are aggregated as “chemical-shift clusters” in the HCA heatmap, as illustrated in Figure 2. The deeper the yellow colour in the map, the higher the intensity of ^13^C NMR peaks.

The NMR chemical shift values for each cluster were submitted to our internal natural product database, comprising predicted NMR data for natural products (*n* ≈ 8500 records in July 2023). Database proposals were then systematically examined for all atom positions by rigorously scrutinizing experimental values, proton/proton, and proton/carbon correlations from the spectra of CPC fractions (^1^H, ^13^C, HSQC, HMBC, and COSY spectra). When experimental data did not exactly match with predicted data, the chemical structures detected in the extract were further elucidated by the manual interpretation of the NMR data (Supplementary Data). The NMR results were also cross-checked with LC–MS analyses to reinforce the identification process (Table 1).

Clusters 1, 2, and 3 were observed in fractions F01–F02 and were assigned to the non-glycosylated flavonoids quercetin (1), naringenin (2), and kaempferol (3), respectively. LC–MS analysis also revealed molecular ions at *m*/*z* 301.0348, *m*/*z* 271.0606, and *m*/*z* 285.0399 at retention times 14.9, 16.1, and 16.2 min, respectively, thereby corresponding to their respective parent ions [M − H]^−^. These three flavonoids are well-known metabolites of *F. ulmaria* flower extracts [31,32]. Cluster 4 corresponded to *β*-sitosterol (4), a very common sterol occurring in many plant species. Clusters 5 and 6 were pentacyclic triterpenes, which were unambiguously confirmed as ursolic acid (5) and pomolic acid (6) by the manual interpretation of 2D NMR data. These two triterpenes were previously reported in the roots and aerial parts of *F. ulmaria* [33]. Cluster 7 corresponded to a group of intense NMR chemical shifts, which were assigned to spiraeoside (7), a biologically active 4′-*O*-monoglycosilated flavonol typically occurring in *F. ulmaria* flowers [34,35]. This compound was largely present in the major fractions F04 and F05 that together represented ≈17% of the crude extract mass, which was identified as an important biomarker of *F. ulmaria*. Its presence was also confirmed by LC–MS with an intense molecular ion *m*/*z* 463.0882 [M − H]^−^ at rt 12.7 min. Cluster 8 corresponded to kaempferol-4′-*O*-glucoside (8), which was also detected in Fractions F04 and F05 but as a minor constituent. This compound was also detected by LC–MS at rt 13.0 min, with *m*/*z* 447.0922 [M − H]^−^. Clusters 9 and 10 were detected in Fractions F02–F03 and were assigned to the phenolic acid derivatives, *p*-anisic acid (9) and ethyl gallate (10). Clusters 11 and 12 were detected in F03–F04 and corresponded to the closely related compounds salicylic acid (11) and salicyl alcohol (12), which are characteristic constituents of the genus *Filipendula* [33,36]. Cluster 13 was detected only in the polar fraction, F10, and assigned to rutoside (13), while Clusters 14 to 16 were assigned to the phenol glycosides, (4-methoxyphenyl)-methyl-glucopyranoside (14), monotropitoside (15), and helicin (16), respectively. The presence of rutoside in the extract was confirmed by LC–MS with the detection of the molecular ion *m*/*z* 609.1450 [M − H]^−^ at rt 11.0 min. Cluster 17 was identified as glycerol (17), while Clusters 18 and 19 detected in the last fraction F13 corresponded to two nucleosides, uridine (18) and adenosine (19), which are universal metabolites distributed in the cells of all living species. Clusters 20 to 25 were unambiguously assigned to monosaccharides and disaccharides, *α*-d-fructofuranose (20), *β*-d-fructopyranose (21), saccharose (22), *β*-d-glucose (23), *β*-d-fructofuranose (24), and α-d-glucose (25). These simple sugars were the major constituents of the most polar fractions, F11–F12, which together represented ~45% of the extract mass. Among sugars, only saccharose was detected by LC–MS at rt 2.2 min, with the corresponding molecular ion observed at *m*/*z* 341.1089 [M-H]^−^. Clusters 26 and 27 were assigned to Tellimagrandin I (26) and Tellimagrandin II (27), which have been described as biologically active constituents of several *Filipendula* species [33,37,38]. These ellagitannins were found to be highly concentrated in fractions, F04–F07, which together represented ≈23% of the dry extract weight and, therefore, were also significant constituents of the extract. Two abundant isomers of Tellimagrandin I were detected by LC–MS in the extract with intense peaks at *m*/*z* 785.0839 and *m*/*z* 785.0840 [M − H]^−^, and rt 8.0 min and 8.9 min, respectively, while Tellimagrandin II was confirmed also by LC–MS with a molecular ion peak at *m*/*z* 937.0955 [M − H]^−^ and rt 10.4 min. Free gallic acid (28) was also identified by NMR in these fractions and detected by LC–MS at rt 5.2 min, with *m*/*z* 169.0137 [M − H]^−^.

**Figure 3:**
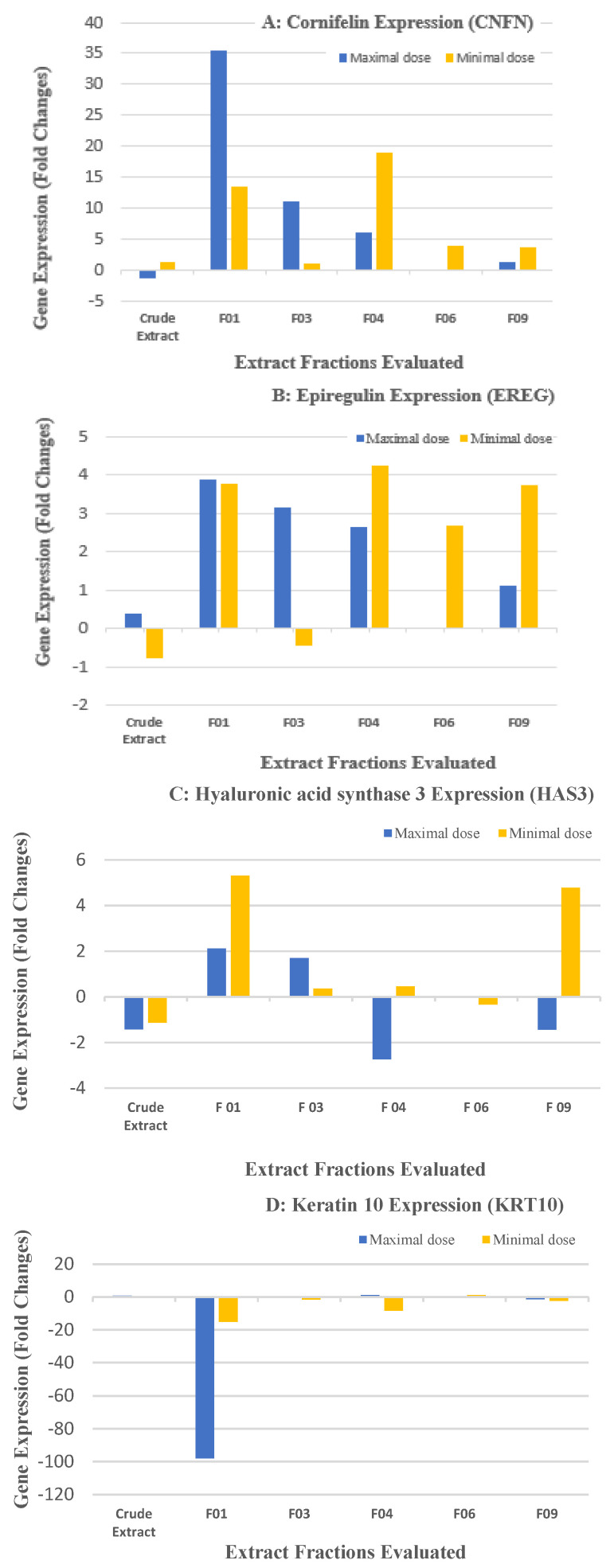

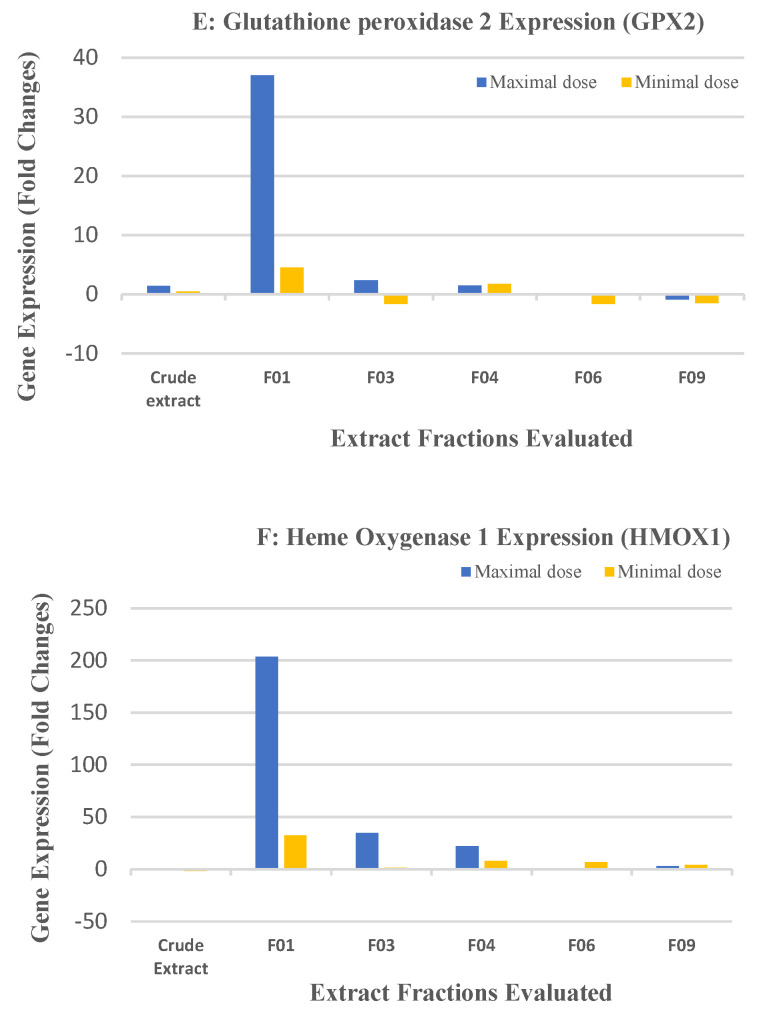
Effect of *F. ulmaria* base extract, Fractions F01, F03, F04, F06, and F09 on epidermal barrier function, epidermal renewal, keratinocyte differentiation, and stress response: (**A**) RT-qPCR on CNFN*; (**B**) RT-qPCR on EREG*; (**C**) RT-qPCR on HAS3*; (**D**) RT-qPCR on KRT10*; (**E**) RT-qPCR on GPX2*; and (**F**) RT-qPCR on HMOX1*. * All evaluations were performed in duplicates.

To summarize, a total of 28 metabolites were unambiguously identified in the *F. ulmaria* extract using the NMR-based CARAMEL dereplication platform. Additional minor metabolites were detected by LC–MS in the extract (Figure 3), including, for instance, several isomers of mono-, di- and tri-galloyl hexosides, several glycosylated derivatives of quercetin and kaempferol, quinic acid, and syringic acid, as well as a diversity of tannins tentatively assigned to chebulagic acid, casuarinin, Rugosin A, and Rugosin D in accordance with the literature related to *F. ulmaria*.

### 2.2. Biological Results for the Crude Extract and CPC Fractions and Assignment of the Metabolites Responsible for Skin Barrier Function Improvement

In an attempt to further explore the cosmetic applications of *F. ulmaria* on epidermal skin barrier, the crude extract was evaluated for the expression of different sets of genes involved in epidermal proliferation, differentiation, barrier function, epidermal renewal, and stress response in normal human epidermal keratinocytes. The selection of normal human epidermal keratinocytes was conducted to eliminate alterations in gene expression due to cellular senescence induced as a result of prolonged culturing of immortalized cell lines, such as HaCaT [39,40]. The *F. ulmaria* extract was cytotoxic at concentrations above 0.002 g/L and was, therefore, tested at the maximal safe concentration of 0.002 g/L and at a lower concentration of 0.0004 g/L. The extract showed moderate modulation of gene expressions at the selected concentrations. However, several secondary metabolites reported from *F. ulmaria,* such as salicylic acid, quercetin and its glycosides, or tannins, are known to affect the skin barrier function and epidermal regulation. Quercetin is known to downregulate the Epiregulin Growth Factor Receptor (EGFR) expression levels [41,42,43], while kaempferol is known to enhance the Claudin 1 gene expression and enhance the intercellular tight junction capacity in the skin [44]. Naringenin and *β*-sitosterol are involved in Glutathione peroxidase regulation [45,46], while ursolic acid and pomolic acid are known to restore the skin barrier function of the epidermis by preventing the trans-epidermal water loss, differentiation of keratinocytes, regulation of peroxisome proliferator-activated receptor-α, and promoting the synthesis of hyaluronic acid and collagen [47,48]. Tellimagrandins present in *F. ulmaria* extracts were also reported previously for enhancing the cornified envelope formation and fillagrin mRNA expression in the HaCaT cell lines [49].

A total of 13 fractions were obtained from the crude *F. ulmaria* extract. The secondary metabolite distribution in the fractions is illustrated in Figure 1. Fractions F01, F03, F04, F06, and F09 were then subjected to epidermal gene expression-modulation tests in the Normal Human Epidermal Keratinocytes for epidermal proliferation, differentiation, barrier function, epidermal renewal, and stress response. Fractions F02, F05, F07, F08, F10, F11, F12, and F13 were not subjected to these tests due to the paucity of the fractions. Fractions F01, F03, F04, F06, and F09 were evaluated at the maximal concentration of 1 g/L and at the minimal dose of 0.2 g/L; the modulation of gene expressions is shown in Figure 3.

Fraction F01 of the *F. ulmaria* extract (4% of the crude extract mass) was found to be rich in quercetin, naringenin, kaempferol, ursolic acid, pomolic acid, and *β*-sitosterol as major constituents. As expected, this fraction showed strong modulation of cornifelin, a tight junction protein, as well as epiregulin, due to the presence of quercetin [44,47,48,49]. Additionally, it was found to enhance the expression of genes involved in hyaluronic acid synthesis 3 and heme oxygenase 1 at both minimal and maximal concentrations. These results demonstrate the high efficacy of this fraction in reinforcing the skin barrier function and renewing the epidermis. Moreover, Fraction F01 showed a significant upregulation of the Glutathione peroxidase 2 gene, likely due to the presence of naringenin and *β*-sitosterol, which have been reported to be involved in regulating glutathione peroxidase activity [45,46,47]. Fraction F03, which mainly comprises the glycosides of quercetin (Spiraeoside) and kaempferol (kaempferol-4′-*O*-glucoside), along with salicylic acid, salicyl alcohol, ethyl gallate, p-anisic acid, and tellimagrandins, account for approximately 5.7% of the total crude extract of *F. ulmaria*. This fraction shows a similar response in gene expression modulation for barrier function reinforcement and epidermal renewal, similar to F01, but only at a maximal concentration of 1 g/L. Its efficacy at the minimal dose was not significant. Fraction F04, which comprises 8% of the total crude extract, contains spiraeoside and tellimagrandins as major constituents, along with small amounts of salicylic acid, salicyl alcohol, and gallic acid. This fraction exhibited a strong modulation of cornifelin, kallikrein-related peptidase 7, heparin-binding EGF-like growth factor, Keratin 19, and Keratin 10 genes at a lower concentration of 0.2 g/L. No significant activity was observed at the maximal dose for fractions containing spiraeoside and tellimagrandin, suggesting that these compounds are effective in keratinocyte differentiation, as well as in reinforcing and renewing the epidermal barrier. Fractions F06 and F09, which comprise 3% and 2% of the crude extract, respectively, contain mainly tellimagrandins and a mixture of minor ellagitannins. Both fractions demonstrated efficacy in epidermal renewal and a restoration of the barrier function at a lower concentration of 0.2 g/L, indicating that ellagitannins play an important role in their efficacy at lower concentrations. This finding is consistent with previously reported activity of tellimagrandins in promoting the formation of cornified envelope [49], as mentioned earlier.

## 3. Materials and Methods

### 3.1. Materials and Reagents

The aerial parts of *F. ulmaria* were collected in 2019 from Somerset County, United Kingdom and were authenticated by an external taxonomist. A voucher specimen has been deposited at the herbarium facility at L’Oreal (Advanced Research, Bangalore, India) under the voucher specimen number ARI 032063/E/B-1/1. HPLC–MS-grade acetonitrile and methanol were purchased from Merck (Lowe, NJ, USA). Laboratory grade chemicals were obtained from Sigma–Aldrich Chemical Co. (St Louis, MO, USA) and Merck Millipore (Darmstadt, Germany). Milli–Q Integral 15 system (Merck Millipore, Burlington, MA, USA) was used to prepare the HPLC-grade water.

### 3.2. Extraction Procedure

The aerial parts of *F. ulmaria* were powdered using the IKA^®^ Pilotina dry-milling system and sieved through 100-µm mesh to afford a coarse powder. The resulting powder (200 g) was extracted using 70% aqueous ethanol (1:10 *m*/*v*) for three consecutive cycles at 60 °C. After three cycles of extraction, each was filtered using GFD filter, and the filtrate was combined under reduced pressure and concentrated to afford a dry extract with a yield of 60 g, containing 4.5% of Spiraeoside. The solid crude extract was further defatted using petroleum ether at 40 °C for 1 h to afford a dry residue containing 6% Spiraeoside. To this crude extract, 2 L of 10% ethanol were added, and the solution was filtered through hyflo supercell. The subsequent clear filtrate was then passed through a HP–20 macroporous resin and eluted with 10 bed volumes of demineralized water and further eluted using increasing percentages of ethanol ranging from 40% ethanol (5 Bed volume (BV)), 50% ethanol (5 BV), and, finally, 100% ethanol. The 50% eluate was concentrated and dried to afford a dry powder highly enriched in Spiraeoside.

### 3.3. Centrifugal Partition Chromatography (CPC)

CPC was performed on a lab-scale FCPE300^®^ column (Rousselet Robatel Kromaton, Annonay, France) of 301-mL capacity, containing seven circular partition disks and engraved with a total of 231 partition twin cells (~1 mL for each twin cell). The liquid phases were pumped with a KNAUER Preparative 1800 V7115 pump (Berlin, Germany). A two-phase solvent system (3 L in total) was prepared by mixing methyl ter-butyl ether, acetonitrile, and water in the proportions 4/1/5 (*v*/*v*) in a separating funnel. After decantation, the column was filled with the lower phase used as the stationary phase at 50 mL/min and 500 rpm. The column rotation speed was then set at 1200 rpm. The extract (1.015 g) was dissolved in an 80/20 (*v*/*v*) mixture of lower and upper phases and injected into the CPC column with a 35-mL loop. The upper phase used as the mobile phase was pumped at a flow rate of 20 mL/min in the ascending mode for 55 min, and then the column was extruded by switching the mode selection valve for 10 min. Fractions of 20 mL were collected over the whole experiment (elution and extrusion) by a Pharmacia Superfrac collector (Uppsala, Sweden), and combined according to their thin layer chromatography (TLC) profiles. TLC was performed with a CAMAG^®^ Automatic TLC Sampler 4 (ATS4), a CAMAG^®^ Automatic Developing Chamber 2 (ADC2), and a CAMAG^®^ TLC Visualizer 2. Fractions were deposited on pre-coated silica gel 60 F254 Merck plates, eluted with the migration solvent system toluene/ethyl acetate/formic acid/acetic acid (30/70/11/11, *v*/*v*) and revealed at 254 nm, at 360 nm, and by spraying the dried plates with 50% H_2_SO_4_ and vanillin, followed by heating. As a result, 13 final fractions were obtained, and their mass distribution and TLC profile are provided in Figure 1.

### 3.4. NMR Analyses and Metabolite Identification

All CPC fractions, F01–F13, were dried under vacuum with a rotary evaporator. An aliquot (up to 15 mg when possible) was dissolved in 600 µL of DMSO-d6 and analyzed by 1H, ^13^C, HSQC, HMBC, and COSY NMR at 298 K on a Bruker Avance III 600 spectrometer (Karlsruhe, Germany) equipped with a cryogenic probe. The Bruker TopSpin 4.0.5 software was used for NMR data acquisition and processing. For ^13^C NMR analyses, a standard zgpg pulse sequence was used with an acquisition time of 0.9 s and a relaxation delay of 3 s. For each sample, 512 scans were co-added to obtain a satisfactory signal-to-noise ratio. The spectral width was 240 ppm, and the receiver gain was set to the highest possible value. Spectra were manually phased, baseline corrected, and calibrated on the central resonance of DMSO-d_6_ (δ 39.8 ppm). The absolute intensities of ^13^C NMR signals were collected by automatic peak picking, and the peak list obtained for each fraction was exported as a text file. Then, a bucketing was performed using a script written in Python, consisting in the division of ^13^C NMR spectral width into chemical shift buckets of 0.3 ppm and an association of the absolute intensity of each peak to the corresponding bucket. The resulting table was submitted to HCA using the PermutMatrix 1.9.3 software (LIRMM, Montpellier, France) for data visualization. In parallel, a literature survey was performed to obtain the structures of a maximum of metabolites already reported in *F. ulmaria* (*n* ≈ 55). The ^13^C NMR chemical shifts of these metabolites were predicted (NMR Workbook Suite 2012, ACD/Labs, Toronto, ON, Canada) and stored into an in-house database comprising ~8300 chemical structures of natural molecules. The chemical shift clusters obtained by HCA were submitted to this database for metabolite identification. Two-dimensional NMR analyses (HSQC, HMBC, and COSY) were also interpretated to validate or complete the structural elucidation of the metabolites proposed by the database.

### 3.5. Liquid Chromatography—Mass Spectrometry Analyses of the Extract (LC/MS)

**Figure 4:**
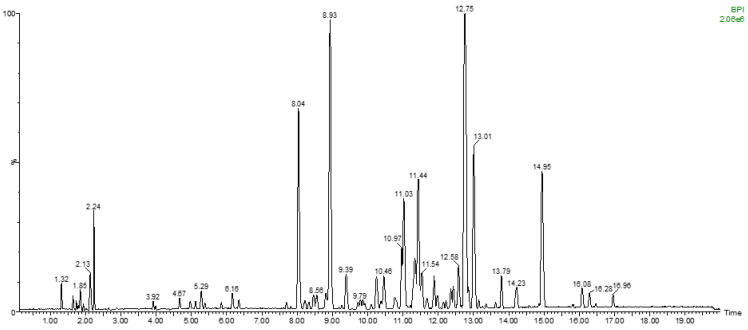
LC/MS chromatogram of the crude extract of *Filipendula ulmaria*.

The crude *F. ulmaria* extract was also analyzed by LC–MS in the negative ion mode to tentatively confirm the identification of a maximum of metabolites. A 5-mg aliquot of each dried fraction was dissolved in 1 mL of MeOH/H_2_O (1:1, *v*/*v*) and analysed on a SYNAPT G2–Si High-Resolution Mass Spectrometer from Waters (St Quentin en Yvelines, France). The chromatographic separation was performed at 1 mL/min on an RP18 reversed-phase column (Uptisphere C-18 ODB 150 × 4.6 mm, 5 µm, Interchim) with an injection volume of 5 µL. The column temperature was maintained at 35 °C. The solvents were formic acid 0.1% in ultrapure water (A) and formic acid 0.1% in LC/MS grade acetonitrile (B). The gradient started at 0% (B), increased up to 26% (B) in 9.9 min to 65% (B) at 18.5 min, and then reached 100% (B) at 18.7 min and remained for 5 min. After that, the gradient returned back to 0% (B) in 1 min and remained for 2 min. The electrospray source operated in the negative mode with the following parameters: capillary voltage 3 kV, sampling cone 40 V, extraction cone 4 V, source temperature 150 °C, desolvation 650 L/h, collision energy 4 V. Accurate mass was ensured using a solution of leu-enkephalin as a standard compound in the internal lockmass. Ions were detected from *m*/*z* 50 to 2000 with scans of 0.2 s. Data were processed with the MassLynx software version 4.2 from Waters. The resulting BPI chromatogram and summarized LC/MS data are provided in Figure 4.

### 3.6. Biological Assays

A Roche LightCycler480 instrument with 384-well microplates was used for this evaluation test of gene expression involved in keratinocytes epidermal physiology. Normal Human Epidermal Keratinocytes were grown and amplified to produce cells for the evaluation. Forty-eight-well microplates were seeded with cells (50,000 cells per well) and incubated for 48 h in a temperature-, humidity-, and CO_2_-controlled environment. Samples were added on the cells while renewing the culture medium and were further incubated for 24 h. After incubation, the cells were washed and frozen at −80 °C to preserve the RNA. The RNAs were then extracted and quantified, and their quality was checked before performing their reverse transcription into cDNA. An RT-qPCR was finally performed for each experimental condition for the quantification of the expression of a set of 16 selected genes related to the barrier function (Claudin 1, Cornifelin, Desmoglein 1, Kallikrein-related peptidase 7, Tight junction protein 1), epidermal renewal (Epiregulin, Hyaluronic acid synthase 3, Heparin-binding EGF-like growth factor, Keratin 19), keratinocyte differentiation (Keratin 10, Small prolin-rich protein A1, Transglutaminase 1), and stress response (Glutathione peroxidase 2, Heme oxygenase 1) in keratinocytes. The gene expression was measured on the highest non-toxic dose of each tested sample with a maximal dose of 0.2 g/L or 0.2 mM. The maximum non-cytotoxic dose was determined prior to the gene-expression testing at a dose of 0.2 g/L using a biological model under the same incubation conditions. All samples were evaluated at the same concentration in addition to a five-times-lower dose in a one-step protocol. The fold changes (FC) were calculated after a double normalization against the housekeeping genes and non-treated condition. Fold changes of gene expression were considered as modulated over 1.5 (induction) or under 0.5 (repression).

## 4. Conclusions

The application of CPC fractionation, ^13^C NMR de-replication, and liquid chromatography hyphenated with mass spectrometry led to the rapid identification of 28 secondary metabolites from the aerial parts of *F. ulmaria*. This study showcased the agile capability of our metabolomic workflow as sensitive and convenient for the chemical profiling of natural resources. This analytical approach offers a disruptive route for discovering and developing new cosmetics with biologically active secondary metabolites from *F. ulmaria*. By subjecting CPC fractions of *F. ulmaria* to an in vitro screening, quercetin, kaempferol glycosides, ursolic acid, pomolic acid, naringenin, *β*-sitosterol, and Tellimagrandins I and II were identified as key secondary metabolites involved in upregulated genes related to the skin-barrier function, epidermal renewal, and stress responses in normal human epidermal keratinocytes. This research could provide a natural solution for improving skin hydration with epidermal-renewal properties. This new screening strategy, without the need for any purification step, should find widespread application where plants have not been widely studied for the discovery of new natural products, as these platforms serve as a future strategic discovery tool.

## Figures and Tables

**Table 1:** Overview of LC/MS data. The crude extract of *Filipendula ulmaria* was analyzed in the negative ion mode. * Also identified by NMR using the CARAMEL approach.

Retention Time (rt in min)	Observed *m*/*z*	Molecular Formula	Δppm	Tentative Identification
1.6	285.0815	C_9_H_17_O_10_	−2.5	Not assigned
1.8	195.0505 [M − H]^−^	C_6_H_11_O_7_	0.0	Hexonic acid
1.9	191.0555 [M − H]^−^	C_7_H_11_O_6_	−0.5	Quinic acid
2.2	341.1089 [M − H]^−^	C_12_H_21_O_11_	1.5	Saccharose *
4.6	331.0664 [M − H]^−^	C_13_H_15_O_10_	0.0	Mono-*O*-galloyl-hexoside isomer 1
4.9	331.0665 [M − H]^−^	C_13_H_15_O_10_	−0.3	Mono-*O*-galloyl-hexoside isomer 2
5.1	339.1292	C_13_H_23_O_10_	0.3	Not assigned
5.2	169.0137 [M − H]^−^	C_7_H_5_O_5_	0.0	Gallic acid *
5.4	483.0783 [M − H]^−^	C_20_H_19_O_14_	1.7	Di-*O*-galloyl-hexoside isomer 1
5.8	331.0667 [M − H]^−^	C_13_H_15_O_10_	0.6	Mono-*O*-galloyl-hexoside isomer 3
6.1	483.0775 [M − H]^−^	C_20_H_19_O_14_	0.2	Di-*O*-galloyl-hexoside isomer 2
6.3	315.0715 [M − H]^−^	C_13_H_15_O_9_	−0.3	Dihydroxybenzoic acid *O*-hexoside
7.7	319.0423 [M − H]^−^	C_15_H_11_O_8_	−9.7	Dihydromyricetin
7.8	483.0775 [M − H]^−^	C_20_H_19_O_14_	0.0	Di-*O*-galloyl-hexoside isomer 3
8.0	785.0839 [M − H]^−^	C_34_H_25_O_22_	0.3	Tellimagrandin I * or isomer
8.2	635.0889 [M − H]^−^	C_27_H_23_O_18_	0.8	Tri-*O*-galloyl-hexoside
8.3	451.1010 [M − H]^−^	C_24_H_19_O_9_	−4.2	Coumaroylepigallocatechin
8.4	375.0694 191.0556 quinic acid fragment	C_18_H_15_O_9_ C_7_H_11_O_6_	−5.9 0.0	Not assigned
8.5	289.0714 909.0999, 785.0842, 454.0461	C_15_H_13_O_6_	0.7	Catechin
8.8	953.0895 [M − H]^−^ 909.0999 [M − COOH]^−^ 785.0837, 465.0367, 454.0460	C_41_H_29_O_27_ C_40_H_29_O_25_ C_34_H_25_O_22_	−0.1 0.0	Chebulagic acid or isomer
8.9	785.0840 [M − H]^−^	C_34_H_25_O_22_	0.4	Tellimagrandin I * or isomer
9.3	319.0431	C_15_H_11_O_8_		Not assigned
9.7	339.0718	C_15_H_15_O_9_	0.6	Not assigned
9.8	359.0745 337.0925 coumaroylquinic acid 191.0556 quinic acid fragment	C_18_H_15_O_8_ C_16_H_17_O_8_ C_7_H_11_O_6_		Not assigned
9.9	785.0845 [M − H]^−^ 481.1118, 491.1403, 625.1407	C_34_H_25_O_22_	1.0	Minor isomer of Tellimagrandin I
10.1	935.0803 [M − H]^−^ 467.0357 [M − H-3galloyl]^−^	C_41_H_27_O_26_	1.3	Casuarinin or Casuarictin
10.2	1105.1012 [M − H]^−^ 1061.1110 fragment of Rugosin D 936.0874 [M − 2H]^2−^ 530.0513 fragment of rugosin A 541.0423	C_48_H_33_O_31_ C_47_H_33_O_29_	0.5 0.2	Rugosin A Rugosin D
10.4	937.0955 [M − H]^−^ 959.0774, 479.0345, 468.0435	C_41_H_29_O_26_	0.9	Tellimagrandin II *
10.8	935.0800 [M − H]^−^ 787.1003 [M − H-galloyl]^−^ 467.0357 [M − H-3galloyl]^−^ 303.0485 [M − H-4galloyl]^−^	C_41_H_27_O_26_	1.0	Casuarinin or Casuarictin
10.9	687.3029 [M − H]^−^	xx	xx	Not assigned
11.0	609.1450 [M − H]^−^	C_27_H_29_O_16_	−1.0	Rutoside *
11.3	197.0454 [M − H]^−^	C_9_H_9_O_5_	2.0	Syringic acid
11.4	463.0877 [M − H]^−^	C_21_H_19_O_12_	0.0	Quercetin *O*-hexoside isomer 1
11.5	463.0876 [M − H]^−^ 301.0348 quercetin fragment	C_21_H_19_O_12_	−0.2	Quercetin *O*-hexoside isomer 2
11.9	593.1505 [M − H]^−^ 1087.0900 [2M − H]^−^ 285.0396 kaempferol fragment	C_27_H_29_O_15_	−0.2	Kaempferol-*O*-hexoside-rhamnoside
11.9	433.0771 [M − H]^−^	C_20_H_17_O_11_	0.0	Quercetin-*O*-pentoside
12.1	447.0930 [M − H]^−^	C_21_H_19_O_11_	0.0	Quercetin-*O*-rhamnoside
12.2	477.1034 [M − H]^−^	C_22_H_21_O_12_	0.2	Methyl-quercetin *O*-hexoside
12.3	433.0772 [M − H]^−^ 301.0353 quercetin fragment	C_20_H_17_O_11_	0.2	Quercetin-*O*-pentoside
12.4	447.0927 [M − H]^−^	C_21_H_19_O_11_	0.0	Quercetin-*O*-rhamnoside
12.5	477.1031 [M − H]^−^	C_22_H_21_O_12_	−0.4	Methylquercetin-*O*-hexoside
12.7	463.0882 301.0353 quercetin fragment	C_21_H_19_O_12_	1.1	Spiraeoside * (Quercetin-*O*-hexoside isomer 3)
12.8	601.0827 [M − H]^−^ 301.0347 quercetin fragment	C_27_H_21_O_16_	−0.5	Quercetin-*O*-galloyl-pentoside
13.0	447.0922 [M − H]^−^ 285.0389 kaempferol fragment	C_21_H_19_O_11_	−1.1	Kaempferol-4′-O-glucoside *
13.2	519.1136 465.1031	C_24_H_23_O_13_ C_21_H_21_O_12_	−0.6 −0.4	Not assigned
13.4	615.0984 [M − H]^−^	C_28_H_23_O_16_	−0.3	Quercetin-*O*-galloyl-hexoside
13.8	585.0880 [M − H]^−^ 301.0350 quercetin fragment	C_27_H_21_O_15_ C_15_H_9_O_7_	0.9 0.7	Quercetin-*O*-galloyl-arabinoside
14.2	297.0399 [M − H]^−^	C_16_H_9_O_6_	−7.1	Not assigned
14.9	301.0348 [M − H]^−^	C_15_H_9_O_7_	1.0	Quercetin *
16.1	271.0606 [M − H]^−^	C_15_H_11_O_5_	0.4	Naringenin *
16.2	285.0399 [M − H]^−^	C_15_H_9_O_6_	1.1	Kaempferol *
16.5	329.2329	C_18_H_33_O_5_	0.3	Tri-HOME (trihydroxyyoctadecenoic acid)
16.9	287.222	C_16_H_31_O_4_	1.5	Dihydroxypalmitic acid

## Data Availability

Not applicable.

## Supplementary Materials

The following supporting information can be downloaded at: https://www.mdpi.com/article/10.3390/molecules28176349/s1.

## Sample Availability

Samples of the compounds are available from the authors.

## Abbreviations

CPC	Centrifugal Partition Chromatography
NMR	Nuclear Magnetic Resonance
HCA	Hierarchical Clustering Analysis
BV	Bed Volume
TLC	Thin Layer Chromatography
HSQC	Heteronuclear single quantum coherence
HMBC	Heteronuclear multiple-bond coherence
COSY	Correlated Spectroscopy
LC-MS	Liquid Chromatography Mass Spectra
FC	Fold Changes
CLDN1	Claudin 1
CNFN	Cornifelin
DSG1	Desmoglein 1
KLK7	Kallikrein-related peptidase 7
TJP1	Tight junction protein 1
EREG	Epiregulin
HAS3	Hyaluronic acid synthase 3
HBEGF	Heparin-binding EGF-like growth factor
KRT19	Keratin 19
KRT10	Keratin 10
SPRR1A	Small prolin-rich protein A1
TGM1	Transglutaminase 1
GPX2	Glutathione peroxidase 2
HMOX1	Heme oxygenase 1
PPAR	Peroxisome proliferator-activated receptor-α

## References

[1] Ball P.W., Miller I.N., Tutin T.G., Heywood V.H., Burges N.A., Moore D.M., Valentine D.H., Walters S.M., Webb D.A. (1969). Flora Europaea.

[2] Šarić-Kundalić B., Dobeš C., Klatte-Asselmeyer V., Saukel J. (2011). Ethnobotanical survey of traditionally used plants in human therapy of east, north and north-east Bosnia and Herzegovina. J. Ethnopharmacol..

[3] Vogl S., Picker P., Mihaly-Bison J., Fakhrudin N., Atanasov A.G., Heiss E.H., Wawrosch C., Reznicek G., Dirsch V.M., Saukel J. (2013). Ethnopharmacological in vitro studies on Austria’s folk medicine—An unexplored lore in vitro anti-inflammatory activities of 71 Austrian traditional herbal drugs. J. Ethnopharmacol..

[4] Halkes S.B.A. (1998). *Filipendula ulmaria*—A Study on the Immunomodulatory Activity of Extracts and Constituents. Ph.D. Thesis.

[5] (1983). British Herbal Pharmacopoeia.

[6] (1990). British Herbal Pharmacopoeia.

[7] Bradley P.R. (1992). British Herbal Compendium.

[8] Bespalov V.G., Alexandrov V.A., Semenov A.L., Kovan’ko E.G., Ivanov S.D., Vysochina G.I., Kostikova V.A., Baranenko D.A. (2016). The inhibitory effect of meadowsweet (*Filipendula ulmaria*) on radiation-induced carcinogenesis in rats. Int. J. Radiat. Biol..

[9] Zeylstra H. (1998). *Filipendula* *ulmaria*. Br. J. Phytother..

[10] (2003). Filipendulae ulmaria Herba—Meadowsweet, ESCOP Monographs, European Scientific Cooperative on Phytotherapy, Editor, Thieme. https://escop.com/downloads/meadowsweet/.

[11] Berardesca E., Distante F., Vignoli G., Oresajo C., Green B. (1997). Alpha hydroxyacids modulate stratum corneum barrier function. Br. J. Dermatol..

[12] Bashir S., Dreher F., Chew A., Zhai H., Levin C., Stern R., Maibach H. (2005). Cutaneous bioassay of salicylic acid as a keratolytic. Int. J. Pharm..

[13] Ademola J., Frazier C., Kim S.J., Theaux C., Saudez X. (2002). Clinical evaluation of 40% urea and 12% ammonium lactate in the treatment of xerosis. Am. J. Clin. Dermatol..

[14] Wolfender J.-L., Marti G., Thomas A., Bertrand S. (2015). Current approaches and challenges for the metabolite profiling of complex natural extracts. J. Chromatogr. A.

[15] Koehn F.E., Carter G.T. (2005). The evolving role of natural products in drug discovery. Nat. Rev. Drug Discov..

[16] Yang Z., Wu Y., Zhou H., Cao X., Jiang X., Wang K., Wu S. (2015). A novel strategy for screening new natural products by a combination of reversed-phase liquid chromatography fractionation and ^13^C NMR pattern recognition: The discovery of new anti-cancer flavone dimers from *Dysosma versipellis* (Hance). RSC Adv..

[17] Hubert J., Nuzillard J.-M., Renault J.-H. (2017). Dereplication strategies in natural product research: How many tools and methodologies behind the same concept?. Phytochem. Rev..

[18] Yang J.Y., Sanchez L.M., Rath C.M., Liu X., Boudreau P.D., Bruns N., Glukhov E., Wodtke A., de Felicio R., Fenner A. (2013). Molecular Networking as a Dereplication Strategy. J. Nat. Prod..

[19] Chao R., Hou X.-M., Xu W.-F., Hai Y., Wei M.-Y., Wang C.-Y., Gu Y.-C., Shao C.-L. (2020). Targeted Isolation of Asperheptatides from a Coral-Derived Fungus Using LC-MS/MS-Based Molecular Networking and Antitubercular Activities of Modified Cinnamate Derivatives. J. Nat. Prod..

[20] Ding W., Tian D., Chen M., Xia Z., Tang X., Zhang S., Wei J., Li X., Yao X., Wu B. (2023). Molecular Networking-Guided Isolation of Cyclopentapeptides from the Hydrothermal Vent Sediment Derived Fungus *Aspergillus pseudoviridinutans* TW58-5 and Their Anti-inflammatory Effects. J. Nat. Prod..

[21] Bruguière A., Derbré S., Dietsch J., Leguy J., Rahier V., Pottier Q., Bréard D., Suor-Cherer S., Viault G., Le Ray A.-M. (2020). MixONat, a Software for the Dereplication of Mixtures Based on ^13^C NMR Spectroscopy. Anal. Chem..

[22] Pannakal S.T., Eilstein J., Prasad A., Ekhar P., Shetty S., Peng Z., Bordier E., Boudah S., Paillat L., Marrot L. (2021). Comprehensive characterization of naturally occurring antioxidants from the twigs of mulberry (*Morus alba*) using on-line high-performance liquid chromatography coupled with chemical detection and high-resolution mass spectrometry. Phytochem. Anal..

[23] Hubert J., Nuzillard J.-M., Purson S., Hamzaoui M., Borie N., Reynaud R., Renault J.-H. (2014). Identification of Natural Metabolites in Mixture: A Pattern Recognition Strategy Based on ^13^C NMR. Anal. Chem..

[24] Abedini A., Colin M., Hubert J., Charpentier E., Angelis A., Bounasri H., Bertaux B., Kotland A., Reffuveille F., Nuzillard J.-M. (2020). Abundant Extractable Metabolites from Temperate Tree Barks: The Specific Antimicrobial Activity of *Prunus avium* Extracts. Antibiotics.

[25] Mahdi D.H., Hubert J., Renault J.-H., Martinez A., Schubert A., Engel K.M., Koudogbo B., Vissiennon Z., Ahyi V., Nieber K. (2020). Chemical Profile and Antimicrobial Activity of the Fungus-Growing Termite Strain *Macrotermes bellicosus* Used in Traditional Medicine in the Republic of Benin. Molecules.

[26] Angelis A., Hubert J., Aligiannis N., Michalea R., Abedini A., Nuzillard J.-M., Gangloff S.C., Skaltsounis A.-L., Renault J.-H. (2016). Bio-Guided Isolation of Methanol-Soluble Metabolites of Common Spruce (*Picea abies*) Bark by-Products and Investigation of Their Dermo-Cosmetic Properties. Molecules.

[27] Darme P., Spalenka J., Hubert J., Escotte-Binet S., Debelle L., Villena I., Sayagh C., Borie N., Martinez A., Bertaux B. (2022). Investigation of Antiparasitic Activity of 10 European Tree Bark Extracts on Toxoplasma gondii and Bioguided Identification of Triterpenes in *Alnus glutinosa* Barks. Antimicrob. Agents Chemother..

[28] Hubert J., Kotland A., Henes B., Poigny S., Wandrey F. (2022). Deciphering the Phytochemical Profile of an Alpine Rose (*Rhododendron ferrugineum* L.) Leaf Extract for a Better Understanding of Its Senolytic and Skin-Rejuvenation Effects. Cosmetics.

[29] Favre-Godal Q., Hubert J., Kotland A., Garnier D., Beaugendre C., Gourguillon L., Urbain A., Lordel-Madeleine S., Choisy P. (2022). Extensive Phytochemical Assessment of *Dendrobium fimbriatum* Hook (Orchidaceae). Nat. Prod. Commun..

[30] Grigolon G., Nowak K., Poigny S., Hubert J., Kotland A., Waldschütz L., Wandrey F. (2023). From Coffee Waste to Active Ingredient for Cosmetic Applications. Int. J. Mol. Sci..

[31] Bijttebier S., Van der Auwera A., Voorspoels S., Noten B., Hermans N., Pieters L., Apers S. (2016). A First Step in the Quest for the Active Constituents in *Filipendula ulmaria* (Meadowsweet): Comprehensive Phytochemical Identification by Liquid Chromatography Coupled to Quadrupole-Orbitrap Mass Spectrometry. Planta Medica.

[32] Gainche M., Ogeron C., Ripoche I., Senejoux F., Cholet J., Decombat C., Delort L., Berthon J.-Y., Saunier E., Chezet F.C. (2021). Xanthine Oxidase Inhibitors from *Filipendula ulmaria* (L.) Maxim. and Their Efficient Detections by HPTLC and HPLC Analyses. Molecules.

[33] Farzaneh A., Hadjiakhoondi A., Khanavi M., Manayi A., Bahramsoltani R., Kalkhorani M. (2022). *Filipendula ulmaria* (L.) Maxim. (Meadowsweet): A Review of Traditional Uses, Phytochemistry and Pharmacology. Res. J. Pharmacogn..

[34] Samardžić S., Arsenijević J., Božić D., Milenković M., Tešević V., Maksimović Z. (2018). Antioxidant, anti-inflammatory and gastroprotective activity of *Filipendula ulmaria* (L.) Maxim. and *Filipendula vulgaris* Moench. J. Ethnopharmacol..

[35] Katanić J., Boroja T., Mihailović V., Nikles S., Pan S.-P., Rosić G., Selaković D., Joksimović J., Mitrović S., Bauer R. (2016). In vitro and in vivo assessment of meadowsweet (*Filipendula ulmaria*) as anti-inflammatory agent. J. Ethnopharmacol..

[36] Harbourne N., Jacquier J.C., O’Riordan D. (2009). Optimisation of the aqueous extraction conditions of phenols from meadowsweet (*Filipendula ulmaria* L.) for incorporation into beverages. Food Chem..

[37] Nitta Y., Kikuzaki H., Azuma T., Ye Y., Sakaue M., Higuchi Y., Komori H., Ueno H. (2013). Inhibitory activity of *Filipendula ulmaria* constituents on recombinant human histidine decarboxylase. Food Chem..

[38] Pukalskienė M., Venskutonis R., Pukalskas A. (2015). Phytochemical Characterization of *Filipendula ulmaria* by UPLC/Q-TOF-MS and Evaluation of Antioxidant Activity. Rec. Nat. Prod..

[39] Rockwell G., Johnson G., Sibatani A. (1987). In vitro senescence of human keratinocyte cultures. Cell Struct. Funct..

[40] Katayama S., Skoog T., Jouhilahti E.-M., Siitonen H.A., Nuutila K., Tervaniemi M.H., Vuola J., Johnsson A., Lönnerberg P., Linnarsson S. (2015). Gene expression analysis of skin grafts and cultured keratinocytes using synthetic RNA normalization reveals insights into differentiation and growth control. BMC Genom..

[41] Jung J.H., Lee J.O., Kim J.H., Lee S.K., You G.Y., Park S.H., Park J.M., Kim E.-K., Suh P.-G., An J.K. (2010). Quercetin suppresses HeLa cell viability via AMPK-induced HSP70 and EGFR down-regulation. J. Cell. Physiol..

[42] Huang Y.T., Hwang J.J. (1999). Effects of luteolin and quercetin, inhibitors of tyrosine kinase, on cell growth and metastasis-associated properties in A431 cells overexpressing epidermal growth factor receptor. Br. J. Pharmacol..

[43] Cuevas M.J., Tieppo J., Marroni N.P., Tuñón M.J., González-Gallego J. (2011). Suppression of Amphiregulin/Epidermal Growth Factor Receptor Signals Contributes to the Protective Effects of Quercetin in Cirrhotic Rats. J. Nutr..

[44] Kim J., Cho N., Kim E.-M., Park K.-S., Kang Y.W., Nam J.H., Nam M.S., Kim K.K. (2020). Cudrania tricuspidata leaf extracts and its components, chlorogenic acid, kaempferol, and quercetin, increase claudin 1 expression in human keratinocytes, enhancing intercellular tight junction capacity. Appl. Biol. Chem..

[45] Al-Roujayee A.S. (2017). Naringenin improves the healing process of thermally-induced skin damage in rats. J. Int. Med Res..

[46] Ferreira M.S., Lobo J.M.S., Almeida I.F. (2021). Sensitive skin: Active ingredients on the spotlight. Int. J. Cosmet. Sci..

[47] Lim S.W., Hong S.P., Jeong S.W., Kim B., Bak H., Ryoo H.C., Lee S.H., Ahn S.K. (2007). Simultaneous effect of ursolic acid and oleanolic acid on epidermal permeability barrier function and epidermal keratinocyte differentiation via peroxisome proliferator-activated receptor-α. J. Dermatol..

[48] Tan H., Sonam T., Shimizu K. (2017). The Potential of Triterpenoids from Loquat Leaves (*Eriobotrya japonica*) for Prevention and Treatment of Skin Disorder. Int. J. Mol. Sci..

[49] Yin J., Hwang I.H., Lee M.W. (2019). Anti-acne vulgaris effect including skin barrier improvement and 5α-reductase inhibition by tellimagrandin I from *Carpinus tschonoskii*. BMC Complement. Altern. Med..

