# Supplementary material for: Rapid Chemical Profiling of Filipendula ulmaria Using CPC Fractionation, 2-D Mapping of 13C NMR Data, and High-Resolution LC–MS
Source: Molecules. 2023 Aug 30;28(17):6349. doi: 10.3390/molecules28176349 (PMC10489126; doi:10.3390/molecules28176349)
Supplement: Supplementary file 1 [file molecules-28-06349-s001.zip › molecules-2512822-supplementary.pdf]

molecules

MDPI

Rapid Chemical profiling of *Filipendula Ulmaria* using CPC fractionation, 2-D mapping of ^{13}C NMR data and high-resolution LC-MS

Supplementary File: Experimental ^{1}H and ^{13}C NMR chemical shifts for the metabolites identified in the *Filipendula ulmaria* extract

NMR chemical shifts and atom correlations were fully validated after rigorous scrutinization of 1D and 2D NMR spectra (^{1}H, ^{13}C, HSQC, HMBC and COSY)
Spectrometer: Bruker Avance 600 MHz – cryoprobe
Solvent: **DMSO-*d6***
Molecular structure numbering is different from the IUPAC numbering (ACD/Labs software numbering) - Molecular weight was determined with the ACD/Labs software calculator.

Quercetin

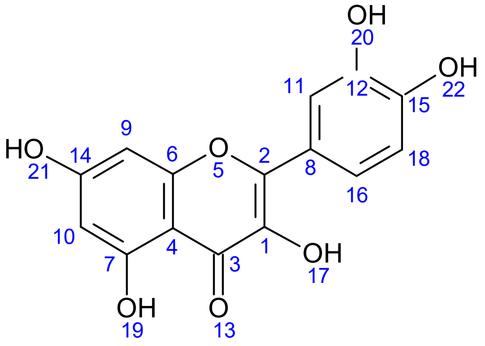

$C_{15}H_{10}O_7$	302.2 g/mol	CAS: 117-39-5
Atom number	^{13}C (ppm)	^{1}H (ppm)
1	136.2	-
2	147.2	-
4	103.4	-
3	176.2	-
6	156.6	-
7	161.2	-
8	122.4	-
9	93.8	6.41
10	98.6	6.18
11	115.5	7.67
12	145.5	-
14	164.3	-

15	148.2	-
16	120.4	7.53
18	116.1	6.89

Naringenin

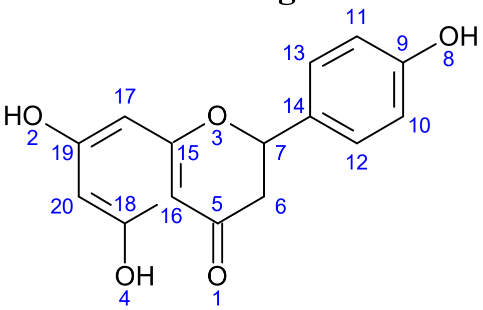

$C_{15}H_{12}O_5$	272.2 g/mol	CAS: 480-41-1
Atom number	^{13}C (ppm)	^{1}H (ppm)
5	196.8	-
6	42.2	2.68/3.27
7	78.9	5.43
9	158.2	-
10/11	115.5	6.80
12/13	128.8	7.33
14	129.4	-
15	Nd*	-
16	102.2	-
17	95.4	5.88
18	163.3	-
19	167.1	-
20	96.2	5.88

Nd* not well-detected

Kaempferol

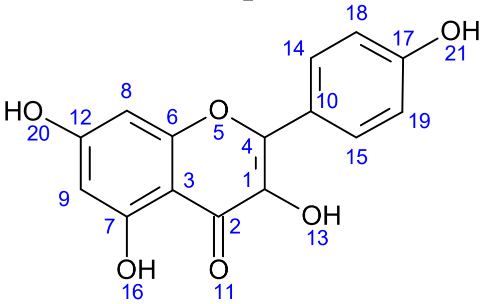

$C_{15}H_{10}O_6$	286.2 g/mol	CAS: 520-18-3
Atom number	^{13}C (ppm)	^{1}H (ppm)
1	136.0	-
2	176.3	-
3	103.3	-
4	147.0	-
6	156.5	-
7	161.1	-
8	93.8	6.44
9	98.6	6.19
10	122.0	-
12	164.3	-
14	129.8	8.04
15	129.8	8.04
17	159.6	-
18	115.8	6.93
19	115.8	6.93

Ursolic acid

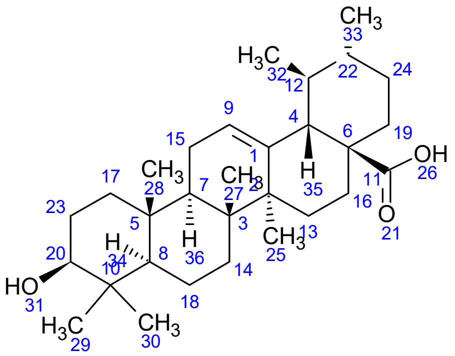

$C_{30}H_{48}O_3$	456.7 g/mol	CAS: 77-52-1
Atom number	^{13}C (ppm)	^{1}H (ppm)
1	138.7	-
2	42.1	-
3	39.5	-
4	52.8	2.10
5	36.9	-
6	47.2	-
7	47.4	1.46
8	55.2	0.67
9	125.0	5.12
10	38.8	-
11	178.6	-
12	38.9	1.29
13	28.0	0.98/1.80
14	33.2	1.26/1.44
15	23.3	1.80/1.84
16	24.2	1.51/1.92
17	38.6	0.90/1.52
18	18.5	1.29/1.46
19	38.5	1.47
20	77.3	2.99
22	38.9	0.92
23	27.5	1.44
24	30.6	1.26/1.41
25	23.6	1.03
27	17.3	0.75
28	15.6	0.85
29	28.7	0.89
30	16.6	0.67
32	17.4	0.80
33	Nd*	Nd*

Pomolic acid

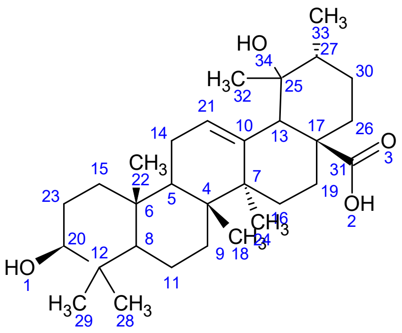

$C_{30}H_{48}O_4$	472.7 g/mol	CAS: 13849-91-7
Atom number	^{13}C (ppm)	^{1}H (ppm)
4	39.6	-
5	47.2	1.46
6	36.8	-
7	41.3	-
8	55.0	0.68
9	32.9	1.25/1.44
10	138.8	-
11	18.2	1.31/1.47
12	38.7	-
13	53.4	2.38
14	23.4	1.85/1.92
15	38.5	0.91/1.53
16	Nd*	Nd*
17	47.2	-
18	16.8	0.71
19	25.5	2.52
20	77.1	3.00
21	127.1	5.16
22	15.4	0.86
23	27.2	1.45
24	24.2	1.29
25	71.9	-
26	Nd*	Nd*
27	41.7	1.26
28	16.2	0.68
29	28.6	0.89
30	26.1	1.13/1.62
31	179.2	-
32	26.6	1.08
33	16.5	0.84

Nd*not well detected

β-sitosterol

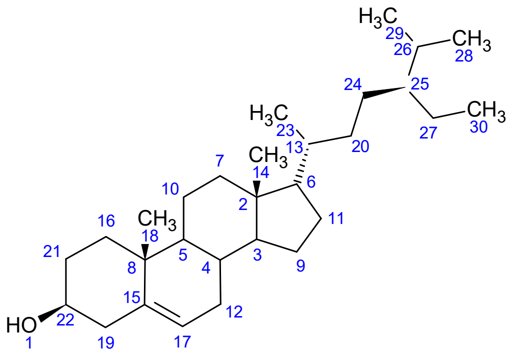

$C_{29}H_{50}O$	414.7 g/mol	CAS: 83-46-5
Atom number	^{13}C (ppm)	^{1}H (ppm)
2	42.0	-
3	56.4	0.95
4	31.7	1.38
5	50.0	0.86
6	55.9	1.07
7	39.5	1.11/1.94
8	36.3	-
9	30.8	1.49/1.80
10	20.7	1.38/1.46
11	27.9	1.28/1.85
12	31.5	1.49/1.93
13	35.9	1.33
14	11.9	0.64
15	140.7	-
16	37.1	0.98/1.77
17	121.4	5.32
18	19.3	0.95
19	42.9	2.25/2.39
20	33.6	0.99/1.15
21	29.6	1.48/1.79
22	77.1	3.48
23	18.8	0.90
24	25.7	1.14/1.27
25	45.4	0.91
26	28.9	1.63
27	22.9	1.19/1.26
28	19.1	0.80
29	19.9	0.82
30	12.0	0.82

Spiraeoside

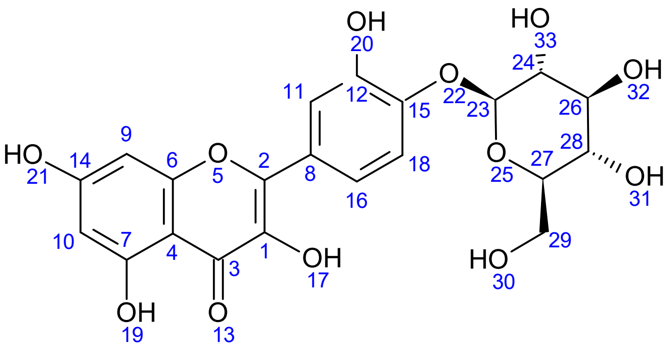

$C_{21}H_{20}O_{12}$	464.4 g/mol	CAS: 20229-56-5
Atom number	^{13}C (ppm)	^{1}H (ppm)
1	136.8	-
2	147.1	-
3	176.5	-
4	103.5	-
6	156.7	-
7	161.1	-
8	125.5	-
9	93.9	6.46
10	98.7	6.21
11	115.5	7.71
12	147.3	-
14	164.4	-
15	146.9	-
16	119.9	7.62
18	116.2	7.26
23	101.8	4.86
24	73.7	3.34
26	76.4	3.32
27	77.7	3.39
28	70.2	3.21
29	61.1	3.50/3.74

Kaempferol 4'-*O*-glucoside

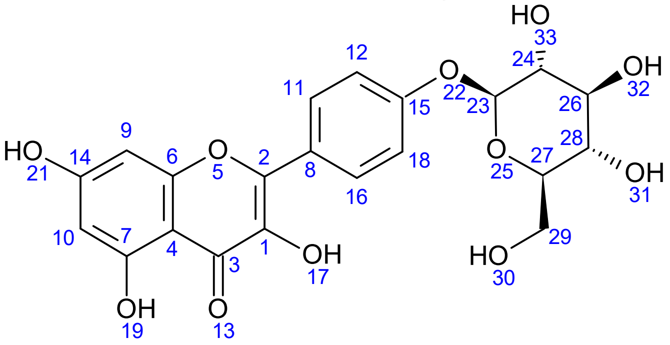

$C_{21}H_{20}O_{12}$	464.4 g/mol	CAS: 20229-56-5
Atom number	^{13}C (ppm)	^{1}H (ppm)
1	136.8	-
2	146.4	-
3	176.4	-
4	103.5	-
6	156.8	-
7	161.4	-
8	124.8	-
9	93.9	6.46
10	98.7	6.21
11	129.6	8.15
12	116.6	7.21
14	164.4	-
15	158.9	-
16	129.6	8.15
18	116.6	7.21
23	100.4	5.00
24	73.7	3.30
26	76.4	3.31
27	77.7	3.39
28	70.2	3.21
29	61.1	3.49/3.74

***p*-anisic acid**

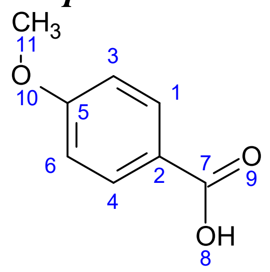

$C_8H_8O_3$	152.1 g/mol	CAS: 100-09-4
Atom number	^{13}C (ppm)	^{1}H (ppm)
1/4	131.8	7.89
2	123.4	-
3/6	114.2	7.02
5	163.3	-
11	56.0	3.82
7	167.5	-

Ethyl gallate

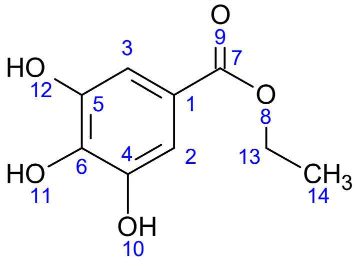

$C_9H_{10}O_5$	198.2 g/mol	CAS: 831-61-8
Atom number	^{13}C (ppm)	^{1}H (ppm)
1	119.5	-
2/3	108.9	6.94
4/5	145.9	-
6	138.8	-
7	166.2	-
13	60.4	4.20
14	14.7	1.27

Salicylic acid

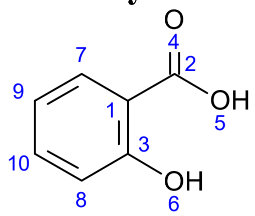

$C_7H_6O_3$	138.1 g/mol	CAS: 69-72-7
Atom number	^{13}C (ppm)	^{1}H (ppm)
1	113.7	-
2	172.4	-
3	161.7	-
7	130.7	7.79
8	117.5	6.93
9	119.5	6.91
10	135.9	7.49

Salicyl alcohol

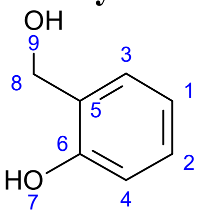

$C_7H_8O_2$	124.1 g/mol	CAS: 90-01-7
Atom number	^{13}C (ppm)	^{1}H (ppm)
1	119.1	6.77
2	127.7	7.04
3	127.8	7.27
4	114.9	6.74
5	128.9	-
6	154.6	-
8	58.6	4.47

Rutoside

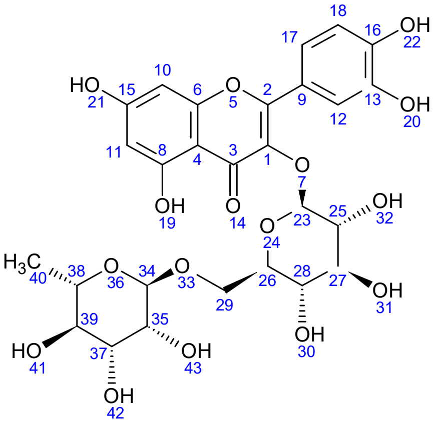

$C_{27}H_{30}O_{16}$	610.5 g/mol	CAS: 153-18-4
Atom number	^{13}C (ppm)	^{1}H (ppm)
1	133.6	-
2	157.0	-
3	177.6	-
4	104.4	-
6	156.8	-
8	161.5	-
9	121.7	-
10	94.0	6.40
11	99.1	6.20
12	116.6	7.53
13	145.1	-
15	164.4	-
16	148.7	-
17	121.9	7.54
18	115.6	6.86
23	101.5	5.34
25	74.3	3.22
26	77.4	3.08
27	76.7	3.21
28	70.5	3.05
29	67.3	3.28/3.70
34	101.1	4.38
35	71.1	3.29
37	70.9	3.39
38	68.6	3.27
39	72.1	3.08
40	18.1	0.99

(4-methoxyphenyl)methyl β-D-glucopyranoside

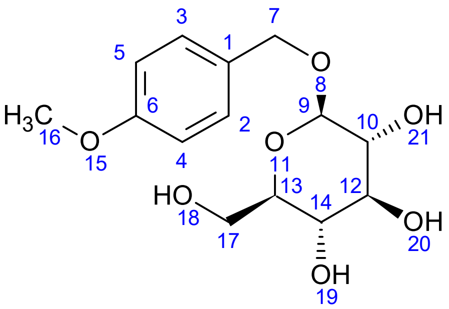

$C_{14}H_{20}O_7$	300.3 g/mol	CAS: 81381-72-8
Atom number	^{13}C (ppm)	^{1}H (ppm)
1	130.4	-
2/3	129.9	7.31
4/5	113.9	6.90
6	159.1	-
16	55.3	3.74
7	69.6	4.50/4.74
9	102.1	4.19
10	74.0	3.01
12	77.2	3.12
14	70.6	3.06
13	77.4	3.09
17	61.6	3.46/3.70

Glycerol

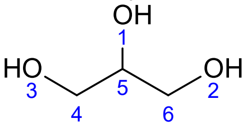

$C_3H_8O_3$	92.1 g/mol	CAS: 56-81-5
Atom number	^{13}C (ppm)	^{1}H (ppm)
5	73.0	3.44
4/6	63.3	3.35/3.28

Monotropitoside

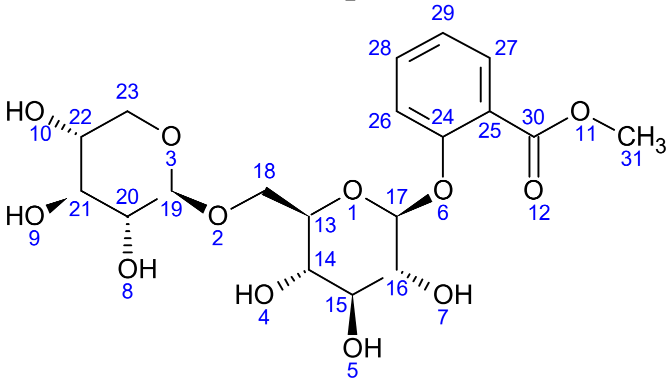

$C_{19}H_{26}O_{12}$	446.4 g/mol	CAS: 490-67-5
Atom number	^{13}C (ppm)	^{1}H (ppm)
13	76.5	3.56
14	70.1	3.18
15	76.8	3.30
16	73.7	3.29
17	101.3	4.86
18	68.6	3.58/3.97
19	104.4	4.19
20	73.9	2.98
21	77.0	3.08
22	70.0	3.25
23	66.1	2.95/3.68
24	156.5	-
25	121.5	-
26	117.0	7.35
27	130.6	7.63
28	134.1	7.53
29	122.1	7.08
30	166.8	-
31	52.6	3.80

Helicin

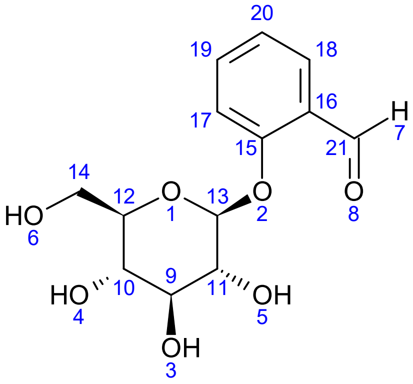

$C_{13}H_{16}O_7$	284.2 g/mol	CAS: 618-65-5
Atom number	^{13}C (ppm)	^{1}H (ppm)
9	77.8	3.62
10	Nd*	Nd*
11	73.6	3.35
12	76.4	3.57
13	101.4	4.95
14	Nd*	Nd*
15	160.3	-
16	125.2	-
17	117.2	7.42
18	127.2	7.70
19	137.0	7.66
20	122.6	7.15
21	190.6	-

Nd* not well detected (or not 100% sure)

Uridine

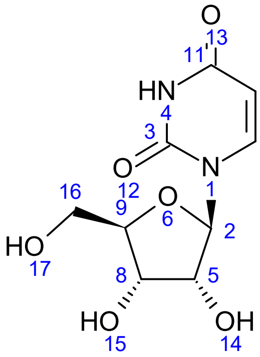

$C_9H_{12}N_2O_6$	244.2 g/mol	CAS: 58-96-8
Atom number	^{13}C (ppm)	^{1}H (ppm)
2	88.0	5.77
3	151.0	-
5	73.9	4.02
7	141.1	7.90
8	70.3	3.95
9	85.1	3.83
10	102.0	5.65
11	163.6	-
16	61.3	3.61/3.55

Adenosine

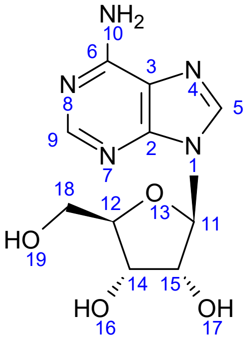

$C_{10}H_{13}N_5O_4$	267.2 g/mol	CAS: 58-61-7
Atom number	^{13}C (ppm)	^{1}H (ppm)
2	149.5	-
3	119.7	-
5	140.4	8.36
6	156.6	-
9	152.9	8.13
11	88.3	5.87
15	74.0	4.59
14	71.1	4.15
12	86.4	3.96
18	62.1	3.55/3.65

Saccharose

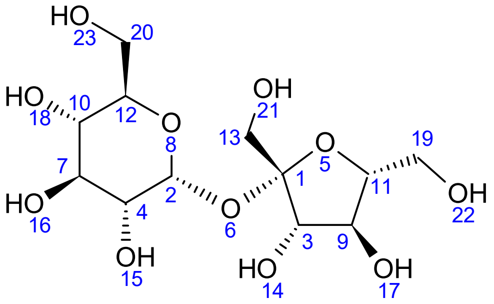

$C_{12}H_{22}O_{11}$	342.3 g/mol	CAS: 57-50-1
Atom number	^{13}C (ppm)	^{1}H (ppm)
1	104.4	-
2	92.1	5.18
3	77.4	3.88
4	71.9	3.18
7	73.2	3.47
9	74.6	3.78
10	70.2	3.12
11	82.9	3.56
12	73.1	3.65
13	62.4	3.40
19	62.5	3.55/3.39
20	60.8	3.49/3.56

α-D-fructofuranose

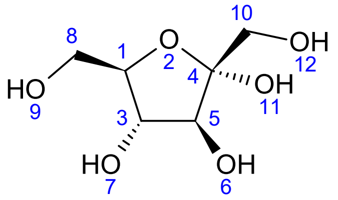

$C_6H_{12}O_6$	180.1 g/mol	CAS: 10489-79-9
Atom number	^{13}C (ppm)	^{1}H (ppm)
1	81.3	3.71
3	76.2	3.74
4	104.5	-
5	83.4	3.80
8	61.6	3.42/3.55
10	64.5	3.42/3.28

β-D-fructofuranose

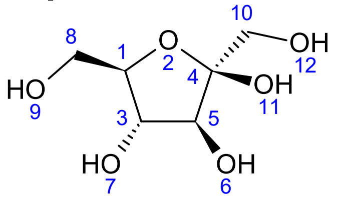

$C_6H_{12}O_6$	180.1 g/mol	CAS: 470-23-5
Atom number	^{13}C (ppm)	^{1}H (ppm)
1	82.3	3.54
3	75.8	3.81
4	102.5	-
5	76.2	3.84
8	63.3	3.40/3.51
10	63.6	3.25/3.38

β-D-fructopyranose

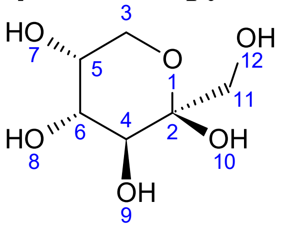

$C_6H_{12}O_6$	180.1 g/mol	CAS: 7660-25-5
Atom number	^{13}C (ppm)	^{1}H (ppm)
2	98.5	-
3	63.5	3.79
4	68.2	3.57
5	69.5	3.65
6	70.2	3.57
11	64.7	3.27/3.43

β-D-glucose

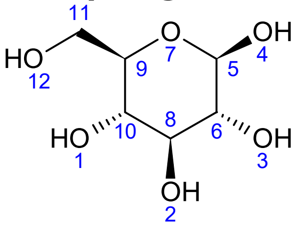

$C_6H_{12}O_6$	180.1 g/mol	CAS: 492-61-5
Atom number	^{13}C (ppm)	^{1}H (ppm)
5	97.2	4.26
6	75.5	2.90
8	77.1	3.04
10	70.9	3.13
9	77.0	3.11
11	61.5	3.65/3.43

α-D-glucose

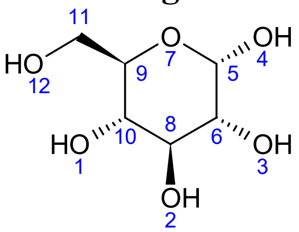

$C_6H_{12}O_6$	180.1 g/mol	CAS: 492-62-6
Atom number	^{13}C (ppm)	^{1}H (ppm)
5	92.6	4.90
6	73.4	3.43
8	72.7	3.11
10	72.3	3.54
9	71.0	3.03
11	61.1	3.55/3.48

Tellimagrandin I

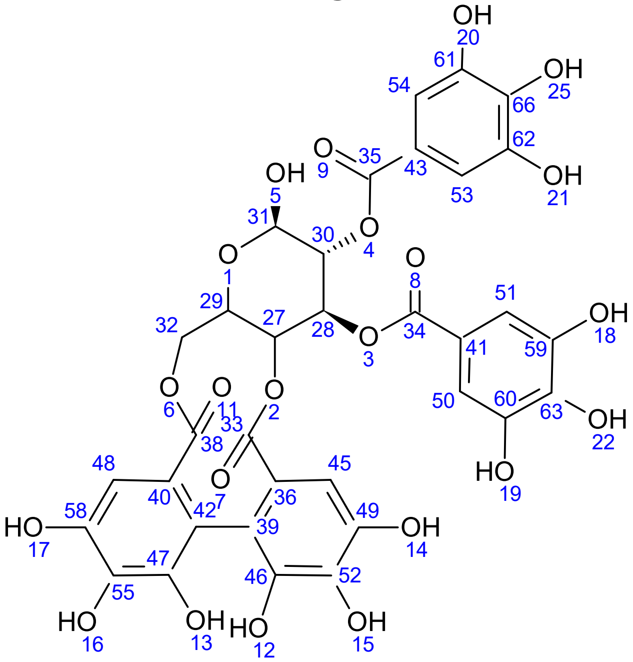

$C_{34}H_{26}O_{22}$	786.5 g/mol	CAS: 79786-08-6
Atom number	^{13}C (ppm)	^{1}H (ppm)
27	70.7	4.90
28	70.4	5.70
29	66.3	4.55
30	72.1	5.01
31	90.2	5.39
32	63.0	3.78/5.12
33	167.2	-
34	165.9	-
35	165.6	-
36	121.0	-
39	115.7	-
40	Nd*	-
45	105.9	6.30
46	Nd*	-
47	Nd*	-
49	144.8	-
52	135.8	-
42	115.8	-
48	106.2	6.43
43	119.0	-
53/54	109.2	6.89
61/62	145.9	-
66	139.3	-
41	118.7	-
50/51	109.2	6.82
59/60	145.8	-

63	139.3	-
55	135.7	-
58	144.8	-

Nd* Not well detected (or not confirmed)

Tellimagrandin II

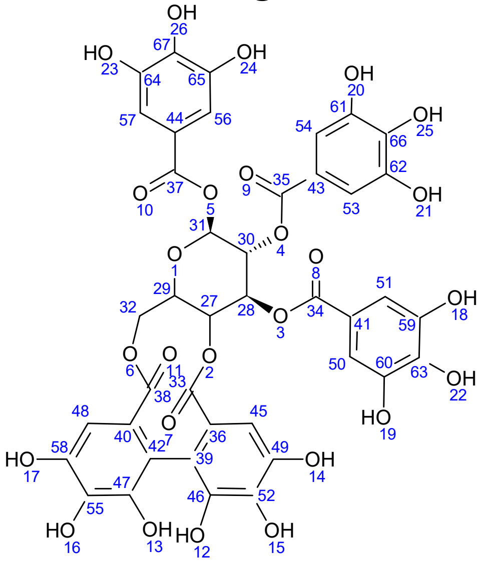

$C_{34}H_{26}O_{22}$	786.5 g/mol	CAS: 79786-08-6
Atom number	^{13}C (ppm)	^{1}H (ppm)
27	70.2	4.96
28	72.2	5.80
29	71.8	4.66
30	71.0	5.40
31	92.3	6.21
32	62.6	3.878/5.12
33	167.2	-
34	165.6	-
35	Nd*	-
36	Nd*	-
37	164.5	-
39	115.6	-
40	Nd*	-
45	105.8	6.28
46	Nd*	-
47	Nd*	-
49	144.8	-
52	135.8	-
42	115.7	-
48	106.2	6.43
43	119.0	-
53/54	Nd*	Nd*
61/62	Nd*	-
66	Nd*	-

41	118.7	-
50/51	109.4	6.99
59/60	145.9	-
63	139.4	-
55	135.7	-
58	144.8	-
56/57	109.4	7.07
64/65	146.1	-
67	139.6	-

Nd* Not well detected (or not confirmed)

Gallic acid

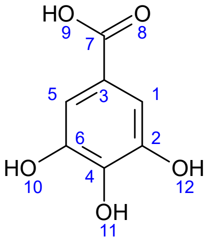

$C_7H_6O_5$	170.5 g/mol	CAS: 149-91-7
Atom number	^{13}C (ppm)	^{1}H (ppm)
1	109.0	6.92
2	145.8	-
3	120.7	-
4	138.3	-
5	109.0	6.92
6	145.8	-
7	167.8	-
